# A Comparative Morphometric and Histological Study of Human Fetus and Fetal Pancreas in Hyperglycemic and Normoglycemic Mothers

**DOI:** 10.7759/cureus.33008

**Published:** 2022-12-27

**Authors:** Rashmi Malhotra, Bharti Jakhar, Kanchan Bisht, Ravi Kant, Ashok Singh, Kavita Khoiwal, Brijendra Singh

**Affiliations:** 1 Anatomy, All India Institute of Medical Sciences, Rishikesh, Rishikesh, IND; 2 Anatomy, All India Institute of Medical Sciences Rishikesh, Rishikesh, IND; 3 General Medicine, All India Institute of Medical Sciences, Rishikesh, Rishikesh, IND; 4 College of Nursing, All India Institute of Medical Sciences, Rishikesh, Rishikesh, IND; 5 Pathology/Histopathology/Renal Pathology, All India Institute of Medical Sciences, Rishikesh, Rishikesh, IND; 6 Obstetrics and Gynecology, All India Institute of Medical Sciences, Rishikesh, Rishikesh, IND

**Keywords:** parameters, gestational age, histogenesis, macrosomic, gestational diabetes mellitus

## Abstract

Background: A significant percentage of pregnancies with gestational diabetes mellitus (GDM) has been found to result in the delivery of macrosomic babies. The current study intends to highlight the correlation between maternal diabetes and fetal parameters as well as the histogenesis of the fetal pancreas in humans.

Materials and methods: The study included thirty aborted fetuses, categorized into seven groups according to their gestational age. Morphometric analysis of fetal parameters and fetal pancreas was done, and the values were compared within different gestational age groups. Pancreatic tissue was processed, stained with Hematoxylin & Eosin, and examined. A comparison was then made between fetuses with and without gestational diabetes.

Results: All the fetal biometrics as well as pancreatic parameters showed greater numeric values in mothers with GDM as compared to the controls of the same gestational age groups. However, the difference was not statistically significant. Histogenesis in such fetuses revealed GDM-related hyperplasia of islets of Langerhans.

Conclusion: A timely diagnosis of GDM is thus of paramount significance due to its potential implications so that appropriate interventions can be done on time, to improve the overall outcome.

## Introduction

The knowledge of gestational age and assigning an accurate expected date of delivery (EDD) is crucial for planning proper antenatal care, as well as improving the overall outcome [1]. The gestational age of the fetus can be calculated by ultrasonography, fetal parameters and different software currently used in this field. Among the various fetal parameters, crown-rump length (CRL) is considered ideal for calculating the gestational age in the first trimester of pregnancy [1-4]. However, due to the variable position of the fetus with advancing gestational age, its accuracy drops owing to the difficulty in measuring the complete length of the fetus [4]. Other fetal parameters like head circumference, biparietal diameter (BPD), abdominal circumference (AC), and femoral diaphysis length (FL), are used for predicting gestational age in the second trimester [5].

When measured separately, AC appears to be a more reliable criterion for the assessment of fetal growth compared to BPD or FL [6,7]. The head circumference (HC) and AC are almost equal at around 34-36 weeks of gestational age, after which the latter gradually exceeds [8]. For the assessment of adequate growth, measuring estimated fetal weight (EFW) can be more helpful rather than any of the above-mentioned parameters [9,10].

Gestational diabetes mellitus (GDM), as described by the American Diabetes Association, is a condition of elevated blood glucose level, detected for the first time in mid or third trimester of pregnancy, without any known cause [11-14]. Increased maternal age as well as weight during pregnancy are among the cardinal risk factors responsible for increasing trend in the global incidence of GDM [15]. Approximately 15%-45% of pregnancies in diabetic mothers result into delivery of macrosomic babies, i.e., babies weighing more than 4,000 grams or more than 90th percentile for their gestational age [16]. In contrast, some diabetic mothers have been reported to give birth to babies with intrauterine growth restriction (IUGR), even though the incidence was lesser than that of macrosomia [8].

During 15th week of gestation, elevated glucose in maternal blood can easily pass down to fetal blood stimulating the pancreatic endocrine component and resulting into hypertrophy of fetal islets with insulin hypersecretion [17,18]. B-cell content in a normal pancreas rises during fetal life, peaks at two months after birth, and then gradually scales down throughout childhood [19,20].

Past studies have been done on animal models, but studies on human pancreatic development are limited. The current study intends to highlight the correlation between maternal diabetes and fetal parameters, as well as histogenesis of fetal pancreas in humans.

## Materials and methods

Study design and participants

Our study is a descriptive observational study. The study included total 30 fetuses, which were aborted spontaneously or by induction in the Obstetrics and Gynecology Department of the Institute.

Data collection

The overall time period for this study was 18 months. The fetuses were collected from the Department after obtaining proper consent and noting down detailed history.

Inclusion criteria

The study included fetuses belonging to the gestational age of 12- 40 weeks, as estimated by the last menstrual period of the mother as well as ultrasonography. We have included the fetuses above 12 weeks of gestation, based on the work by Wirdnam and Milner [19], who have attempted to document the development of the A and B cell fractions in the human pancreas from week 12 of fetal life to puberty.

Exclusion criteria

We ruled out fetuses below 12 weeks of gestational age and those with the malformed pancreas.

Sample size calculation

Assuming the expected population standard deviation to be 6, and employing t-distribution to estimate sample size, the study would require a sample size of 30 to estimate a mean with 95% confidence and a precision of 2.3. In other words, if you select a random sample of 30 from a population, and determine the mean to be Y, you would be 95% confident that the mean in the population lies somewhere between Y - 2.3 and Y + 2.3 [21].

Ethical committee approval

Approval for the present research was granted by the Institutional Ethics Committee (IEC no: AIIMS/IEC/21/485) and clinical trial registration was obtained (Registration no: ECR/736/Inst/UK/2015/RR-21). The present study conforms to the principles of the Declaration of Helsinki.

Data management and statistical analysis

10% formalin, dissection instruments like scalpel, forceps and scissors, measuring instruments like nylon thread, measuring scale, Vernier calipers and weighing machine were used in the study.

The study included a total of thirty fetuses, which were aborted spontaneously or by induction in the Obstetrics and Gynecology Department of the Institute. Seven groups were made for the fetuses according to their gestational: 1st group included fetuses belonging to 12th -16th weeks of gestation, 2nd Group: 17th-20th weeks, 3rd Group: 21st-24th weeks, 4th Group: 25th-28th weeks, 5th Group: 29th-32nd weeks, 6th Group: 33rd-36th weeks and 7th Group included fetuses belonging to 37th-40th weeks of gestation, as shown in Table 1.

**Table 1:** Division of fetal groups according to gestational age.

Group	Gestational week	No. of fetuses
1^st^	12^th^ -16^th^	5
2^nd^	17^th^ -20^th^	6
3^rd^	21^st^-24^th^	6
4^th^	25^th^ -28^th^	2
5^th^	29^th^ -32^nd^	6
6^th^	33^rd^ -36^th^	4
7^th^	37^th^ -40^th^	1
8^th^	Total	30

**Figure 1:**
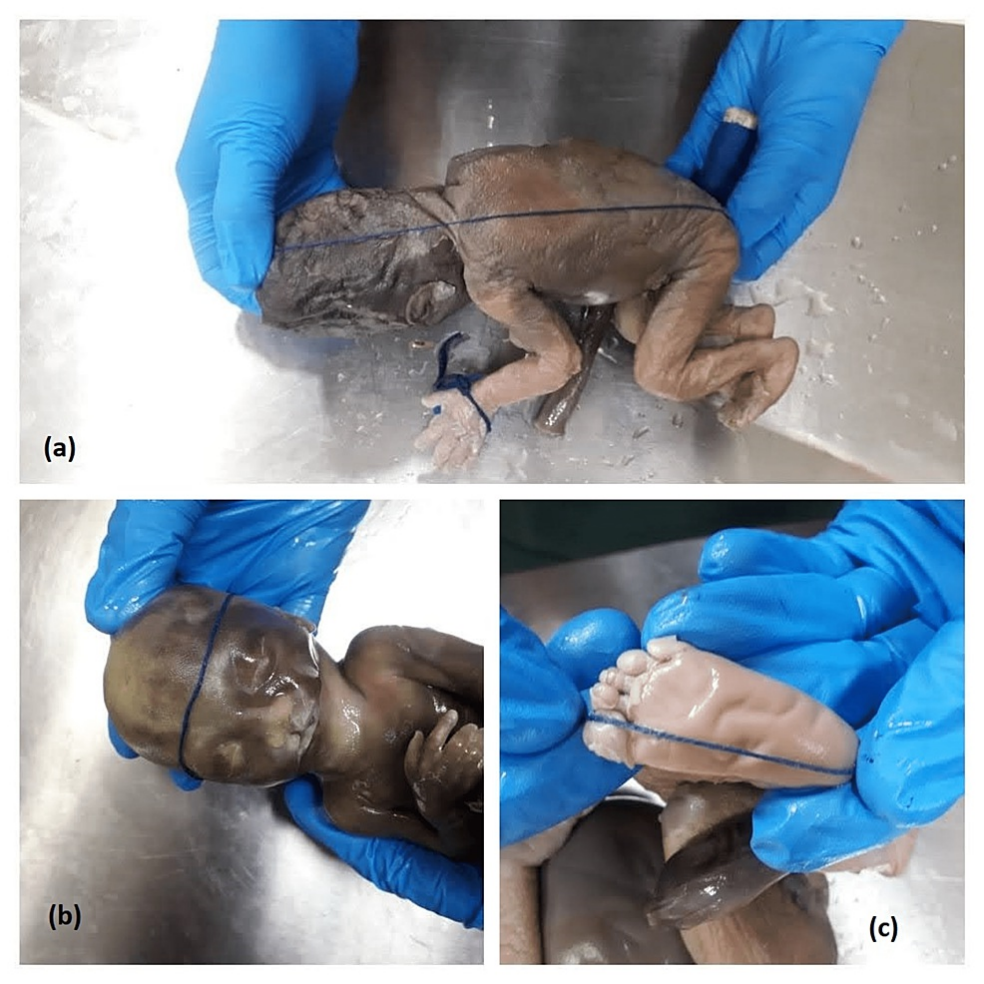
(a-c) Measurement of crown rump length (CRL), head circumference (HC) and foot length (FL), respectively.

Fetuses were collected in 10% formalin immediately after abortion and medical termination of pregnancy. Parameters of fetuses such as sex, weight and gestational age were recorded. Weight in grams was recorded with the help of electronic weighing machine. Morphometric parameters of fetuses like CRL, crown heel length (CHL), head circumference, abdomen circumference, chest circumference, hand length and foot length in centimeters were measured by nylon thread, as illustrated in Figures 1a-1c.

**Figure 2:**
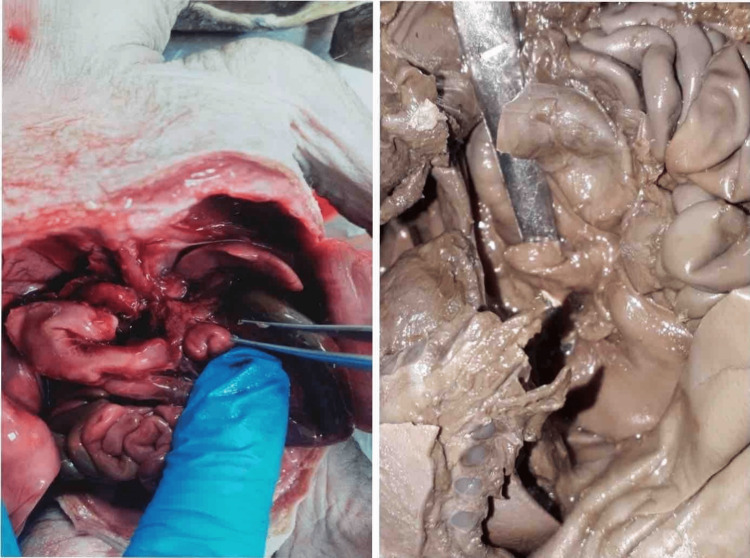
Stepwise dissection (a) opening up of abdominal cavity and (b) dissecting out pancreas.

Following proper steps of dissection, pancreases were taken out from fetuses as illustrated in Figures 2a, 2b.

**Figure 3:**
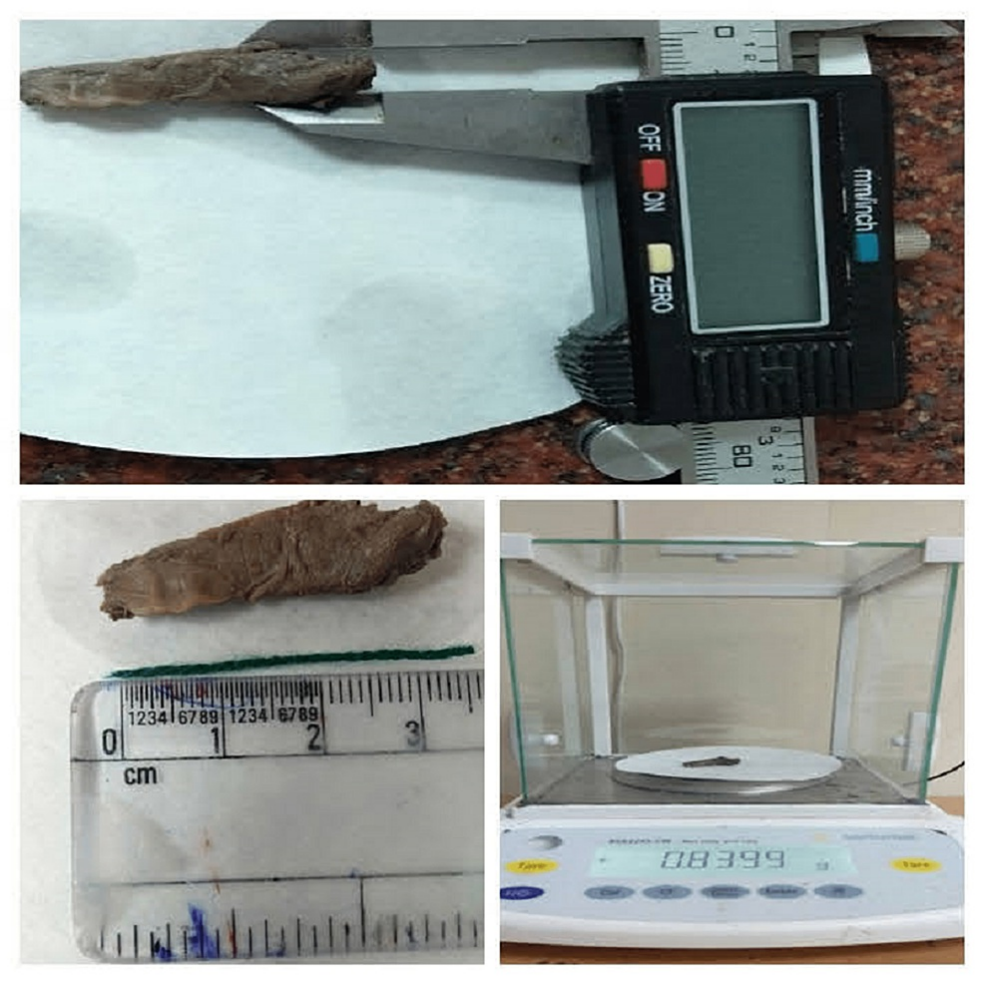
Measurement of different parameters of pancreas.

A midline incision was made from the jugular notch to the pubic symphysis. The pancreas was identified and removed from abdominal cavity. Parameters such as weight, length, and thickness were measured by Vernier calipers (Figure 3) and tabulated.

All these parameters were compared within gestational age groups. Pancreatic tissue was processed, stained with Hematoxylin and Eosin stains and examined. All the values were compared between fetuses with and without maternal GDM.

## Results

Out of total 30 fetuses, four fetuses had mothers with diabetic history, as shown in Table 2.

**Table 2:** Distribution of the participants in terms of diabetic history of mother (n = 30).

Diabetic history of mother	Frequency	Percentage	95% CI (Confidence Interval)
GDM (Gestational Diabetes Mellitus)	4	13.3%	4.4% - 31.6%
NIL	26	86.7%	68.4% - 95.6%

Table 3 represents the association between the diabetic history of the mother and different parameters. All fetal parameters show greater values in mothers with GDM as compared to the controls (mothers without GDM) of the same gestational age group in the later gestational age group of fetuses, although the gap was not significant statistically.

**Table 3:** Association between diabetic history of mother and parameters. ***Significant at p<0.05, 1: Wilcoxon-Mann-Whitney U Test

Parameters	Diabetic History of Mother		P-value
	GDM (Gestational Diabetes Mellitus) (n = 4)	Nil (n = 26)	
CRL (Crown Rump Length)	31.02 ± 11.65	23.03 ± 7.50	0.170^1^
CHL (Crown Heel Length)	52.55 ± 22.43	37.73 ± 13.88	0.222^1^
Abdominal Circumference	21.12 ± 6.86	16.25 ± 5.15	0.179^1^
Chest Circumference	24.50 ± 8.81	17.45 ± 6.33	0.177^1^
Head Circumference	28.50 ± 11.21	20.11 ± 6.45	0.119^1^
Foot Length	5.95 ± 2.93	4.14 ± 1.91	0.170^1^
Hand Length	4.47 ± 1.69	3.15 ± 1.23	0.135^1^
Pancreas Weight (Gram)	2.35 ± 1.23	1.29 ± 1.04	0.179^1^
Pancreas Length (cm)	2.12 ± 1.19	1.83 ± 0.99	0.427^1^
Pancreas Thickness (cm)	0.68 ± 0.26	0.61 ± 0.22	0.806^1^

We have used Fisher's exact test to find out the association between “Diabetic History of Mother” and “Gender” as more than 20% of the total number of cells had an expected count of less than 5 (Table 4). The difference between all the groups was, however, not significant in terms of gender distribution (χ2 = 1.978, p = 0.287).

**Table 4:** Association between diabetic history of mother and gender (n = 30).

Gender	Diabetic History Of Mother			Fisher's Exact Test	
	GDM (Gestational Diabetes Mellitus)	Nil	Total	χ2	P-value
Male	4 (100.0%)	17 (65.4%)	21 (70.0%)	1.978	0.287
Female	0 (0.0%)	9 (34.6%)	9 (30.0%)		
Total	4 (100.0%)	26 (100.0%)	30 (100.0%)		

The variables on Gestational Age (weeks), and all the fetal parameters were not normally distributed in the two subgroups of the variable Diabetic History of Mother. Thus, non-parametric test (Wilcoxon-Mann-Whitney U Test) was used to make group comparisons. As shown in Table 5, the mean of Gestational Age (Weeks) in the fetuses - with and without maternal Diabetic history - was 29.50 ± 9.15 and 23.77 ± 6.98, respectively.

**Table 5:** Comparison of gestational age in fetuses with and without Diabetic History of Mother (n = 30).

Gestational Age (Weeks)	Diabetic History Of Mother		Wilcoxon-Mann-Whitney U Test	
	GDM (Gestational Diabetes Mellitus)	Nil	W	P-value
Mean (SD)	29.50 (9.15)	23.77 (6.98)	74.000	0.189
Median (IQR)	33 (28-34.5)	23 (19-29)		
Range	16 – 36	12 – 38		

**Figure 4:**
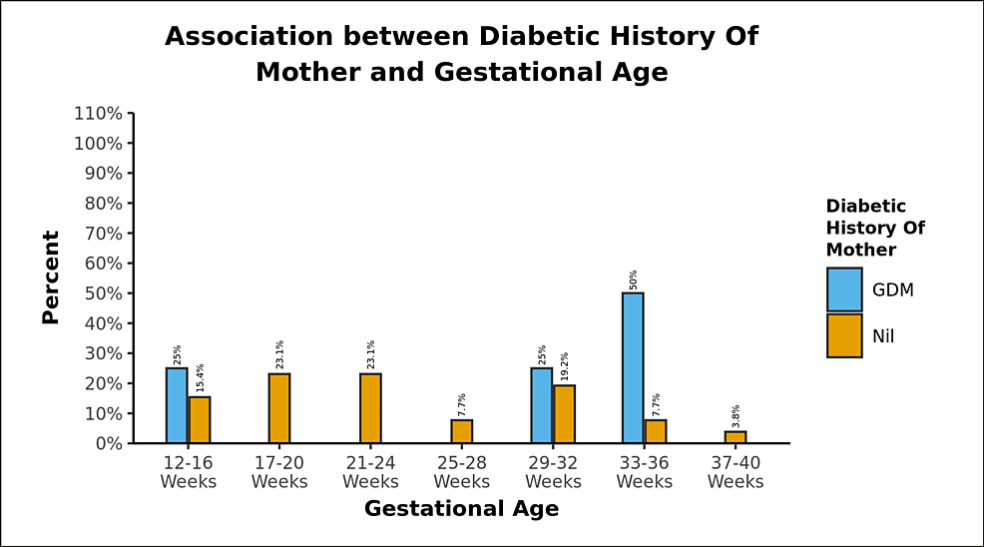
Gestational week-wise distribution of diabetic history of mother.

The difference was again not significant between the various groups in terms of Gestational Age (weeks) (W = 74.000, p = 0.189). 50% of the fetuses with positive history of diabetes in mothers belonged to the Gestational Age: 33-36 weeks, as shown in Figure 4. Early gestational age groups do not show much difference in fetal parameters with regard to gestational diabetes or controls.

As shown in Table 6, the mean of CRL in the fetuses with and without maternal Diabetic History was 31.02 ± 11.65 and 23.03 ± 7.50 respectively. The difference was again not significant in terms of CRL between the groups (W = 75.000, p = 0.170).

**Table 6:** Comparison of crown rump length (CRL) in fetuses with and without diabetic history of (n = 30).

CRL (Crown Rump Length)	Diabetic History Of Mother		Wilcoxon-Mann-Whitney U Test	
	GDM (Gestational Diabetes Mellitus)	Nil	W	P-value
Mean (SD)	31.02 (11.65)	23.03 (7.50)	75.000	0.170
Median (IQR)	34.85 (29.38-36.5)	23.15 (19.5-24.8)		
Range	14 - 40.4	10 - 42.2		

Similarly, the mean of CHL in the two groups was 52.55 ± 22.43 and 37.73 ± 13.88 respectively, as shown in Table 7. Statistically, the difference was not significant between the groups in terms of CHL (W = 72.500, p = 0.222).

**Table 7:** Comparison of CHL (Crown Heel length) in fetuses with and without Diabetic History of Mother (n = 30).

CHL (Crown Heel length)	Diabetic History Of Mother		Wilcoxon-Mann-Whitney U Test	
	GDM (Gestational Diabetes Mellitus)	Nil	W	P-value
Mean (SD)	52.55 (22.43)	37.73 (13.88)	72.500	0.222
Median (IQR)	63.1 (50.8-64.85)	36.05 (31.48-39.73)		
Range	19 - 65	16 - 67.4		

As shown in Table 8, the mean of Abdominal Circumference in the fetuses with and without maternal Diabetic History was 21.12 ± 6.86 and 16.25 ± 5.15, respectively. The difference between the groups was not significant in terms of Abdominal Circumference (W = 74.500, p = 0.179).

**Table 8:** Comparison of abdominal circumference in fetuses with and without diabetic history of mother (n = 30).

Abdominal Circumference	Diabetic History Of Mother		Wilcoxon-Mann-Whitney U Test	
	GDM (Gestational Diabetes Mellitus)	Nil	W	P-value
Mean (SD)	21.12 (6.86)	16.25 (5.15)	74.500	0.179
Median (IQR)	23.75 (20-24.88)	16.4 (14.27-17.45)		
Range	11 - 26	6.2 – 31		

As shown in Table 9, the mean of Chest Circumference in the two groups was 24.50 ± 8.81 and 17.45 ± 6.33, respectively. Again, this difference was not found to be statistically significant between the groups (W = 75.000, p = 0.177).

**Table 9:** Comparison of chest circumference in fetuses with and without diabetic history of mother (n = 30).

Chest Circumference	Diabetic History Of Mother		Wilcoxon-Mann-Whitney U Test	
	GDM (Gestational Diabetes Mellitus)	Nil	W	P-value
Mean (SD)	24.50 (8.81)	17.45 (6.33)	75.000	0.177
Median (IQR)	27 (21.75-29.75)	16.55 (15.38-18.15)		
Range	12 - 32	6.5 - 35.9		

As evident in Table 10, the mean of Head Circumference in the fetuses with and without maternal diabetic history was 28.50 ± 11.21 and 20.11 ± 6.45, respectively. Statistically, the difference was not significant between the groups in terms of Head Circumference (W = 78.000, p = 0.119).

**Table 10:** Comparison of head circumference in fetuses with and without diabetic history of mother (n = 30).

Head Circumference	Diabetic History Of Mother		Wilcoxon-Mann-Whitney U Test	
	GDM (Gestational Diabetes Mellitus)	Nil	W	P-value
Mean (SD)	28.50 (11.21)	20.11 (6.45)	78.000	0.119
Median (IQR)	33.9 (27.97-34.42)	20 (17.15-22)		
Range	11.7 - 34.5	9 – 35		

The mean of Foot length in the two groups, as shown in Table 11, was 5.95 ± 2.93 and 4.14 ± 1.91, respectively. Again, no significant difference was observed between the groups in terms of Foot Length (W = 75.000, p = 0.170).

**Table 11:** Comparison of foot length in fetuses with and without diabetic history of mother (n = 30).

Foot Length	Diabetic History Of Mother		Wilcoxon-Mann-Whitney U Test	
	GDM (Gestational Diabetes Mellitus)	Nil	W	P-value
Mean (SD)	5.95 (2.93)	4.14 (1.91)	75.000	0.170
Median (IQR)	6.85 (4.95-7.85)	4 (2.9-4.85)		
Range	1.8 - 8.3	1.3 - 8.5		

As shown in Table 12, the mean of Hand Length in the fetuses with and without maternal Diabetic history was 4.47 ± 1.69 and 3.15 ± 1.23, respectively. Statistically, the difference was not significant between the groups in terms of Hand Length (W = 77.000, p = 0.135).

**Table 12:** Comparison of hand length in fetuses with and without diabetic history of mother (n = 30).

Hand Length	Diabetic History Of Mother		Wilcoxon-Mann-Whitney U Test	
	GDM (Gestational Diabetes Mellitus)	Nil	W	P-value
Mean (SD)	4.47 (1.69)	3.15 (1.23)	77.000	0.135
Median (IQR)	5.1 (4.1-5.48)	2.95 (2.4-4.15)		
Range	2 - 5.7	0.9 – 6		

Similarly, although the pancreatic parameters were found to increase in fetuses with positive maternal Diabetic history, the values were not statistically different from the other group with negative history of maternal diabetes. As shown in Table 13, the mean of Pancreas Weight (Gram) in the groups with and without maternal history of diabetes was 2.35 ± 1.23 and 1.29 ± 1.04, respectively (W = 74.500, p = 0.179).

**Table 13:** Comparison of pancreas weight in fetuses with and without diabetic history of mother (n = 30).

Pancreas Weight (Gram)	Diabetic History Of Mother		Wilcoxon-Mann-Whitney U Test	
	GDM (Gestational Diabetes Mellitus)	Nil	W	P-value
Mean (SD)	2.35 (1.23)	1.29 (1.04)	74.500	0.179
Median (IQR)	2.8 (1.95-3.2)	0.73 (0.62-1.91)		
Range	0.6 - 3.2	0.4 - 3.5		

As represented in Table 14, the mean of Pancreas Length in the groups with and without maternal history of diabetes was 2.12 ± 1.19 and 1.83 ± 0.99, respectively (W = 65.500, p = 0.427).

**Table 14:** Comparison of pancreas length in fetuses with and without diabetic history of mother (n = 30).

Pancreas Length (cm)	Diabetic History Of Mother		Wilcoxon-Mann-Whitney U Test	
	GDM (Gestational Diabetes Mellitus)	Nil	W	P-value
Mean (SD)	2.12 (1.19)	1.83 (0.99)	65.500	0.427
Median (IQR)	2.42 (1.81-2.72)	1.8 (1.36-2.4)		
Range	0.42 - 3.2	0.2 - 3.7		

The mean of Pancreas Thickness in the two groups was 0.68 ± 0.26 and 0.61 ± 0.22, respectively, as shown in Table 15. Statistically, the difference was not significant between the groups in terms of Pancreas Thickness (W = 56.500, p = 0.806).

**Table 15:** Comparison of pancreas thickness in fetuses with and without diabetic history of mother (n = 30).

Pancreas Thickness	Diabetic History Of Mother		Wilcoxon-Mann-Whitney U Test	
	GDM (Gestational Diabetes Mellitus)	Nil	W	P-value
Mean (SD)	0.68 (0.26)	0.61 (0.22)	56.500	0.806
Median (IQR)	0.7 (0.48-0.9)	0.6 (0.51-0.7)		
Range	0.4 - 0.9	0.2 - 1.3		

**Figure 5:**
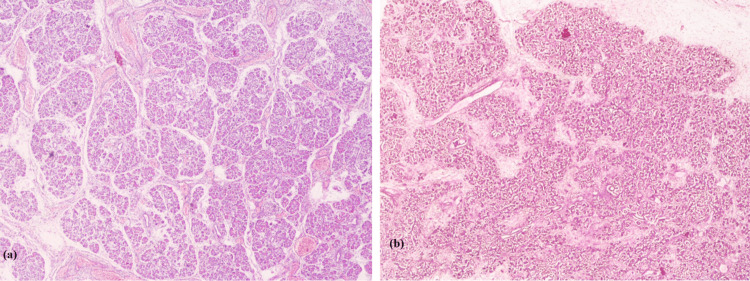
(a) Fetus no. 5 (Non- GDM): H&E-stained pancreas under 4x magnification. (b) Fetus no. 4 (GDM): H&E-stained pancreas under 4x magnification.

Figures 5a, 5b illustrate H&E-stained pancreatic tissue of fetus with and without maternal GDM, respectively.

**Figure 6:**
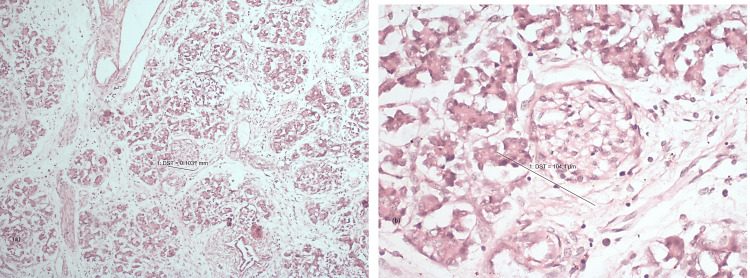
Normal histogenesis in a fetus under (a) 10x and (b) 40x magnification.

**Figure 7:**
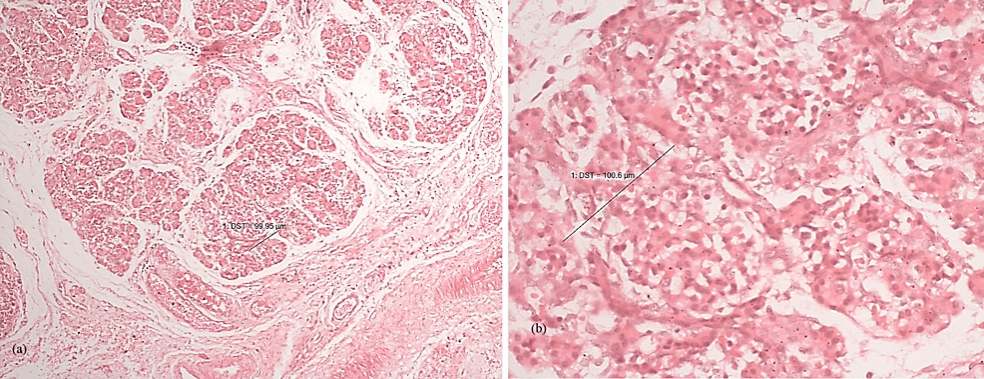
Fetus showing GDM-related hyperplasia of islets of Langerhans under (a) 10x and (b) 40x magnification.

Figures 6a, 6b show normal histogenesis of pancreas under 10x magnification and 40x magnification, respectively, while Figures 7a, 7b represent GDM related hypertrophy of islets of Langerhans under 10x and 40x magnification, respectively.

## Discussion

Approximately 15%-45% of pregnancies in diabetic mothers result in the delivery of macrosomic babies, i.e., babies weighing more than 4,000 grams or more than the 90th percentile for their gestational age [16]. Asian females have been reported to have an increased incidence of GDM, compared to other ethnic groups in the world [22].

The growth pattern in fetuses with and without diabetic mothers remains almost similar up to the beginning of the third trimester, after which slight changes in AC are reported in fetuses of the diabetic mother [8]. This occurs due to uneven accumulation of subcutaneous fat, in such fetuses chiefly in the abdominal and interscapular regions [23].

According to AC were seen during the 32nd to 34th gestational weeks. They observed that fetuses with a maternal history of diabetes showed an accelerated rate of growth, as compared to their counterparts with no such history - especially after the 26th week of gestation. The parameter showing the greatest difference included the abdominal area, femur length as well as biparietal diameter [24]. This was in accordance with our study where all fetal parameters show greater values in mothers with a history of gestational diabetes as compared to the controls of the same gestational age group in a later gestational age group of fetuses, although the difference was not statistically significant.

An ultrasonographic study by Landon et al. revealed growth rate of AC in fetuses with GDM mothers was much higher (1.36 cm/week) in the third trimester when compared to their control counterparts (0.9 cm/week) - even though the growth rate of other parameters in both the groups was more or less the same [25]. However, in our study, the maximum difference was seen in CHL of fetuses with maternal GDM (52.55 ± 22.43) and without GDM (37.73 ± 13.88).

As per the observations made by Brand et al., during the initial phase of pregnancy, fetuses of mothers with GDM showed restricted growth. On the other hand, during the later phase, especially after the 24th week of gestation, the same fetuses showed accelerated growth. AC and EFW were markedly higher in these fetuses in contrast to those without a maternal history of GDM [26].

Rekani et al., in their study, found that babies born to diabetic women weighed more (mean birth weight: 4230 ± 511) than those born to non-diabetic women (mean birth weight: 4053 ± 201) with a statistically significant (p=0.0398) difference. However, their study, along with another one conducted by Saleh et al., reported a higher incidence of macrosomia in babies without diabetic history in mothers, as opposed to those with diabetic mothers [27,28]. The fetal weight was, however, not taken into account in our study for comparison between the fetuses of mothers with and without GDM.

According to former beliefs, in diabetic pregnancy, hypertrophy of islets was thought to be the outcome of elevation in the number of B-cells only. Milner et al. later used immunocytochemical staining methods to show that the elevation in B-cells was associated with hyperplasia of A and PP-cells. It was suggested that diabetes in pregnancy changes the fetal environment influencing the stem cells which are responsible for the development of cells containing pancreatic hormones in the future [29].

The histological study of the pancreas from fetuses of diabetic mothers in our study revealed hyperplasia of islets of Langerhans. Stoyanov et al. reported an autopsy finding of diabetic fetopathy in an aborted fetus, which on a histological study revealed hyperplasia, as well as amyloidosis of Islets of Langerhans. The collection of amyloid proteins was thought to be due to elevated insulin and amylin in the fetus - in response to diabetes in the mother and increased glucose in the fetus [30].

Limitation of study

The present study has a very small sample size, which can limit the generalizability of our findings to a bigger population. So, we would like to propose a bigger study in the future on a larger number of fetuses for a better representation of the findings.

## Conclusions

Our study concluded that with progression of gestational age, all fetal parameters including CRL, CHL, head circumference, abdominal circumference, chest circumference, hand length and foot length, show a higher trend in mothers with gestational diabetes as compared to those without gestational diabetes. A similarly higher trend was observed in pancreatic parameters like length, weight and thickness, of fetuses with maternal history of gestational diabetes as compared to their counterparts whose mothers had no such history. Histogenesis of pancreas in these fetuses revealed hyperplasia of Islets of Langerhans. A timely diagnosis of GDM is thus of paramount significance due to its potential implications so that appropriate interventions can be done on time, improving the overall outcome.

## References

[1] Committee on Obstetric Practice, the American Institute of Ultrasound in Medicine (2017). Committee opinion no 700: methods for estimating the due date. Obstet Gynecol.

[2] Robinson HP, Fleming JE (1975). A critical evaluation of sonar "crown-rump length" measurements. Br J Obstet Gynaecol.

[3] Verburg BO, Steegers EA, De Ridder M (2008). New charts for ultrasound dating of pregnancy and assessment of fetal growth: longitudinal data from a population-based cohort study. Ultrasound Obstet Gynecol.

[4] Hadlock FP, Shah YP, Kanon DJ, Lindsey JV (1992). Fetal crown-rump length: reevaluation of relation to menstrual age (5-18 weeks) with high-resolution real-time US. Radiology.

[5] (2018). AIUM-ACR-ACOG-SMFM-SRU Practice Parameter for the Performance of Standard Diagnostic Obstetric Ultrasound Examinations. J Ultrasound Med.

[6] Kurjak A, Kirkinen P, Latin V (1980). Biometric and dynamic ultrasound assessment of small-for-dates infants: report of 260 cases. Obstet Gynecol.

[7] Weiner CP, Robinson D (1989). Sonographic diagnosis of intrauterine growth retardation using the postnatal ponderal index and the crown-heel length as standards of diagnosis. Am J Perinatol.

[8] Jaffe R (2002). Identification of fetal growth abnormalities in diabetes mellitus. Semin Perinatol.

[9] Dudley NJ, Lamb MP, Hatfield JA, Copping C, Sidebottom K (1990). Estimated fetal weight in the detection of the small-for-menstrual-age fetus. J Clin Ultrasound.

[10] Laurin J, Persson PH (1987). Ultrasound screening for detection of intra-uterine growth retardation. Acta Obstet Gynecol Scand.

[11] American Diabetes Association (2018). 2. Classification and diagnosis of diabetes: standards of medical care in diabetes-2018. Diabetes Care.

[12] Plows JF, Stanley JL, Baker PN, Reynolds CM, Vickers MH (2018). The pathophysiology of gestational diabetes mellitus. Int J Mol Sci.

[13] Baz B, Riveline JP, Gautier JF (2016). Endocrinology of pregnancy: gestational diabetes mellitus: definition, aetiological and clinical aspects. Eur J Endocrinol.

[14] İlhan G, Gültekin H, Kubat A, Gokmen Karasu AF, Güngör ES, Zebitay GA, Verit Atmaca FF (2018). Preliminary evaluation of foetal liver volume by three-dimensional ultrasound in women with gestational diabetes mellitus. J Obstet Gynaecol.

[15] Dubé MC, Girard M, Morisset AS, Tchernof A, John Weisnagel S, Bujold E (2011). Evaluation of fetal liver volume by tridimensional ultrasound in women with gestational diabetes mellitus. J Obstet Gynaecol.

[16] Kc K, Shakya S, Zhang H (2015). Gestational diabetes mellitus and macrosomia: a literature review. Ann Nutr Metab.

[17] Gollin YG, Gracia C, Gollin G, Marks C, Marks W, Papandonatos G (1999). Effect of maternal diabetes on the fetal exocrine pancreas. Early Hum Dev.

[18] Persson B, Heding LG, Lunell NO, Pschera H, Stangenberg M, Wager J (1982). Fetal beta cell function in diabetic pregnancy. Amniotic fluid concentrations of proinsulin, insulin, and C-peptide during the last trimester of pregnancy. Am J Obstet Gynecol.

[19] Wirdnam PK, Milner RD (1981). Quantitation of the B and A cell fractions in human pancreas from early fetal life to puberty. Early Hum Dev.

[20] Stefan Y, Grasso S, Perrelet A, Orci L (1983). A quantitative immunofluorescent study of the endocrine cell populations in the developing human pancreas. Diabetes.

[21] Dhand NK, Khatkar MS (2015). Statulator: An online Statistical calculator. http://statulator.com/SampleSize/ss1M.htm.

[22] Liu B, Lamerato LE, Misra DP (2020). A retrospective analysis of the relationship between race/ethnicity, age at delivery and the risk of gestational diabetes mellitus. J Matern Fetal Neonatal Med.

[23] McFarland MB, Trylovich CG, Langer O (1998). Anthropometric differences in macrosomic infants of diabetic and nondiabetic mothers. J Matern Fetal Med.

[24] Wong SF, Lee-Tannock A, Amaraddio D, Chan FY, McIntyre HD (2006). Fetal growth patterns in fetuses of women with pregestational diabetes mellitus. Ultrasound Obstet Gynecol.

[25] Landon MB, Mintz MC, Gabbe SG (1989). Sonographic evaluation of fetal abdominal growth: predictor of the large-for-gestational-age infant in pregnancies complicated by diabetes mellitus. Am J Obstet Gynecol.

[26] Brand JS, West J, Tuffnell D, Bird PK, Wright J, Tilling K, Lawlor DA (2018). Gestational diabetes and ultrasound-assessed fetal growth in South Asian and White European women: findings from a prospective pregnancy cohort. BMC Med.

[27] Rekani H, Raouf N (2021). Macrosomic infants of diabetic and non-diabetic pregnant women. J PediatrPerinatol Child Health.

[28] Saleh A, Al-Sultan SM, Moria AM, Rakaf FI, Turkistani YM, Al-Onazi SH (2008). Fetal macrosomia greater than or equal to 4000 grams. Comparing maternal and neonatal outcomes in diabetic and nondiabetic women. Saudi Med J.

[29] Milner RD, Wirdnam PK, Tsanakas J (1981). Quantitative morphology of B, A, D, and PP cells in infants of diabetic mothers. Diabetes.

[30] Stoyanov GS, Kobakova I, Stoev L, Popov H, Shishkov SR, Bratoeva K (2019). Histological changes in severe diabetic fetopathy: an autopsy case report. Cureus.

